# Supplementary material for: TRIP13 and APC15 drive mitotic exit by turnover of interphase- and unattached kinetochore-produced MCC
Source: Nat Commun. 2018 Oct 19;9:4354. doi: 10.1038/s41467-018-06774-1 (PMC6195577; doi:10.1038/s41467-018-06774-1)
Supplement: Supplementary file 2 — Supplementary Information [file 41467_2018_6774_MOESM2_ESM.pdf]

TRIP13 and APC15 drive mitotic exit by turnover of interphase- and unattached kinetochore-produced MCC

Kim et al.

Table of Contents

Supplementary Table 1

Supplementary Figure 1

Supplementary Figure 2

Supplementary Figure 3

Supplementary Figure 4

Supplementary Figure 5

Supplementary Table1. Oligonucleotides used in this study

Oligo Name	Sequences
Mad2_H191A_F	CGTTCATTTACTACTACAATCGCCAAAGTAAATAGCATGGTGGC
Mad2_H191A_R	GCCACCATGCTATTTACTTTGGCGATTGTAGTAGTAAATGAACG
TRIP13_E253Q_F	ACTCTCCACCTGATCAATCAGCACGAACACCAG
TRIP13_E253Q_R	CTGGTGTTCGTGCTGATTGATCAGGTGGAGAGT
TRIP13_gRNA	CACCGTCTCGGGGGCGCCATGGACG
Mad2_3'UTR_siRNA1	CCUAUUGAAUCAGUUUCCAAUUU
Mad2_3'UTR_siRNA2	CAGUAUAGGUAGGGAGAUAUU
APC15_siRNA	GUCUGGUCUAAGUUUCUUU
$p31^{comet}$_siRNA	AGTGGTATGAGAAGTCCGAAG
BubR1_UTR_siRNA	CUGUAUGUGCUGUAAUUUAUU
TPR_CDS_siRNA	GCACAACAGGAUAAGGUUAUU
Control siRNA	ON-TARGETplus™ Non-Targeting Pool (Cat# D-001810-10-05)

Supplementary Figure 1. TRIP13 is required for its role in checkpoint activation

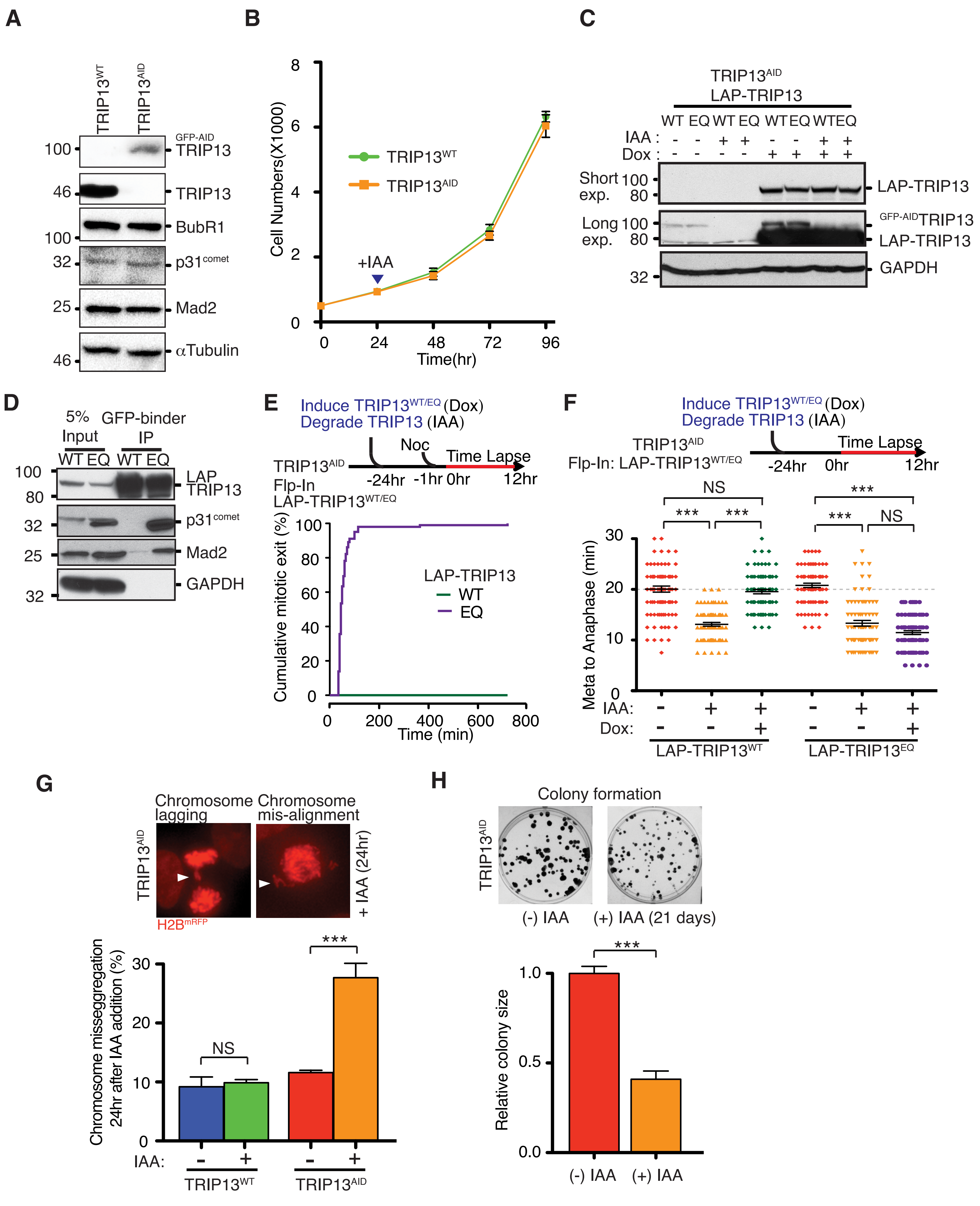

Supplementary Figure 1. TRIP13 ATPase activity is required for checkpoint activation.

A) Protein expression level of TRIP13, BubR1, $p31^{comet}$, Mad2 and α-tubulin were measured by quantitative immunoblotting in $TRIP13^{WT}$ and $TRIP13^{AID}$ cells.

B) Growth rate of $TRIP13^{WT}$ and $TRIP13^{AID}$ in the absence of TRIP13. IAA was added 24 hours after plating.

C) Replacement of GFP-AID-TRIP13 with LAP-$TRIP13^{WT}$ or LAP-$TRIP13^{EQ}$ was monitored by immunoblotting after treatment of IAA and doxycycline in $TRIP13^{AID}$ Flp-In LAP-$TRIP13^{WT/EQ}$ cells.

D) LAP-$TRIP13^{WT}$ or LAP-$TRIP13^{EQ}$ interacting components in $TRIP13^{AID}$. GFP-AID-TRIP13 was replaced with LAP-$TRIP13^{WT}$ or LAP-$TRIP13^{EQ}$ in $TRIP13^{AID}$ Flp-In LAP-$TRIP13^{WT/EQ}$. TRIP13 interacting proteins were immunopurified using GFP-binder beads and analyzed by immunoblotting. The $TRIP13^{EQ}$ mutation stabilizes TRIP13 hexamer formation and inhibits ATP hydrolysis [52], and LAP-$TRIP13^{EQ}$ binds both $p31^{comet}$ and Mad2 more stably than LAP-$TRIP13^{WT}$.

E) TRIP13 ATPase activity is required for mitotic exit. (Top) Schematic of experiments testing the role of TRIP13 ATPase activity in checkpoint activation. (Bottom) Timing of mitotic exit in $TRIP13^{AID}$ Flp-In LAP-$TRIP13^{WT/EQ}$ cells was monitored by live-cell imaging in the presence of nocodazole 24hr after IAA and doxycycline addition (nocodazole was added 1 hour prior to filming). (n=50 for each condition)

F) Mitotic timing after replacement of GFP-AID-$TRIP13^{WT}$ with LAP-$TRIP13^{WT/EQ}$. (n=75 for each condition)

G) *Upper:* Representative images of lagging and mis-aligned chromosomes in $TRIP13^{AID}$ cells after 24-hour IAA treatment. *Lower:* Mitotic error rates of $TRIP13^{WT}$ and $TRIP13^{AID}$ cells after 24-hour IAA treatment were calculated by counting lagging and mis-aligned chromosomes in mitosis. Total cell counted: n=252, 356, 286 and 357 for each experimental condition from 3 independent experiments.

H) *Upper:* Colony size in $TRIP13^{AID}$ cells after 21-day growth with or without IAA. *Lower:* Relative colony size for 21-day IAA treated versus untreated cells (mean ± s.e.m. of six independent

experiments). See Supplementary Figure 1B for analysis of TRIP13^{WT} versus TRIP13^{AID} cell growth in the presence and absence of IAA, and for replacement of TRIP13 with TRIP13^{WT} and TRIP13^{EQ}.

P-values showing in panel F, G and H were calculated in Prism v. 7 using an unpaired two-tailed t-test (**: p<0.01, ***: p<0.001, NS=not significant).

Supplementary Figure 2. Mitotic delay by TRIP13 depletion is dependent on Mad2.

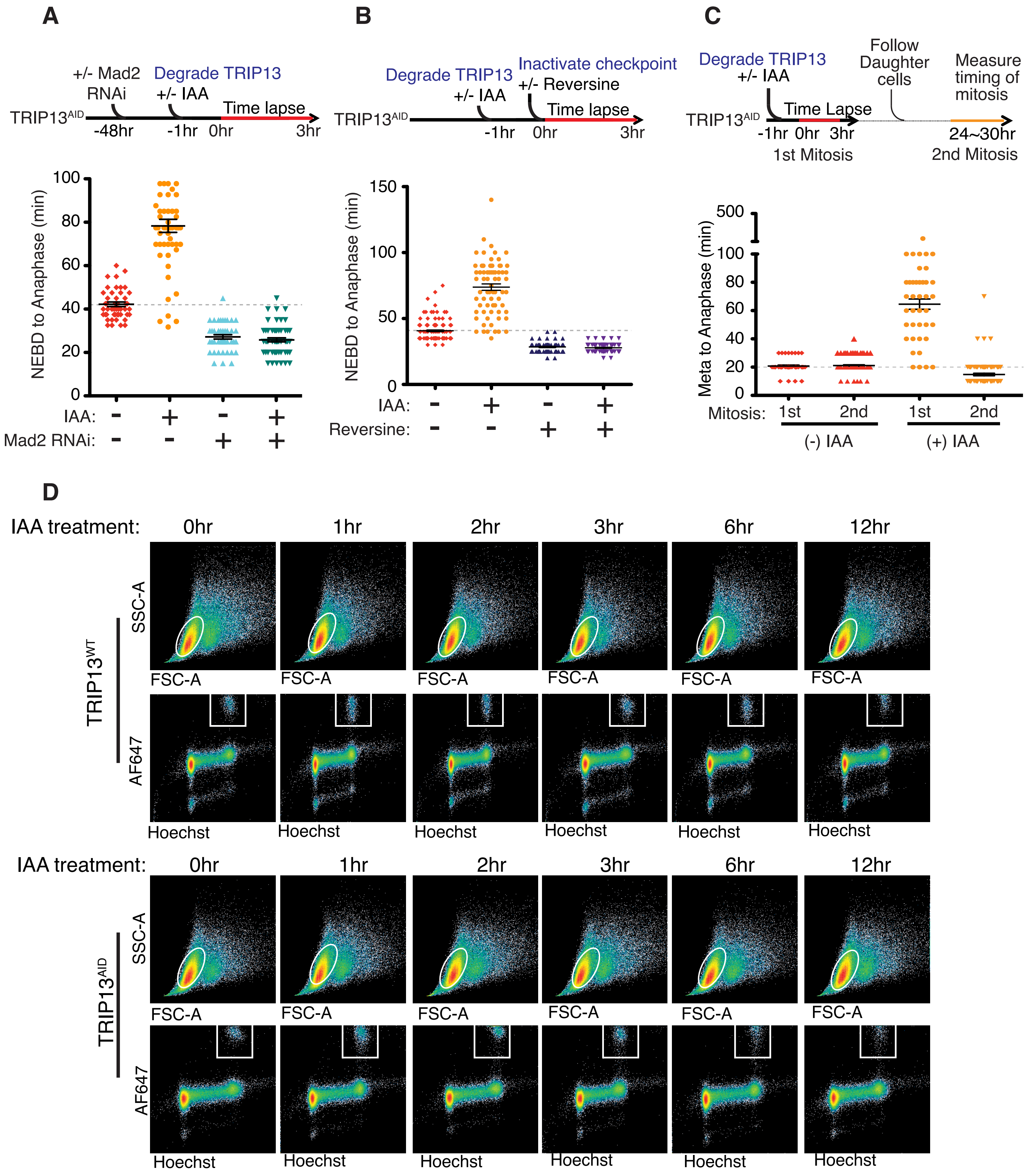

Supplementary Figure 2. TRIP13 regulates checkpoint activation and silencing through MCC.

A) *Upper:* Schematic of experiment to measure the dependence of the observed mitotic exit delay on Mad2. *Lower:* NEBD-to-anaphase timing (there is no definable metaphase in Mad2-depleted cells) in Mad2-RNAi $TRIP13^{AID}$ cells. (n=55 for each condition)

B) *Upper:* Schematic of experiment to measure the dependence of the observed mitotic exit delay on kinetochore-catalyzed MCC production. *Lower:* NEBD-to-anaphase timing in reversine-treated $TRIP13^{AID}$ cells. (From left to right panel, n= 84, 75, 54 and 58)

C) Two rounds of unperturbed cell cycles were monitored after TRIP13 depletion. Cells were strongly delayed in exiting mitosis within the first 3 hours after TRIP13 degradation, but these cells' daughters showed faster-than-wildtype exit from mitosis. The daughter cells likely did not activate the checkpoint for even the short time that most wild-type cells do. (n=50 of parental cells for each condition)

D) Gating strategy to measure mitotic population (Related to Figure 2F). Population of phospho-histone H3 (Ser10) Alexa647 (AF647) positive $TRIP13^{WT}$ and $TRIP13^{AID}$ cells was measured using flow cytometry at different time points after IAA treatment. (Total cells counted for each condition: 10000)

Supplementary Figure 3. TRIP13 alters accumulation of Mad2 and p31.

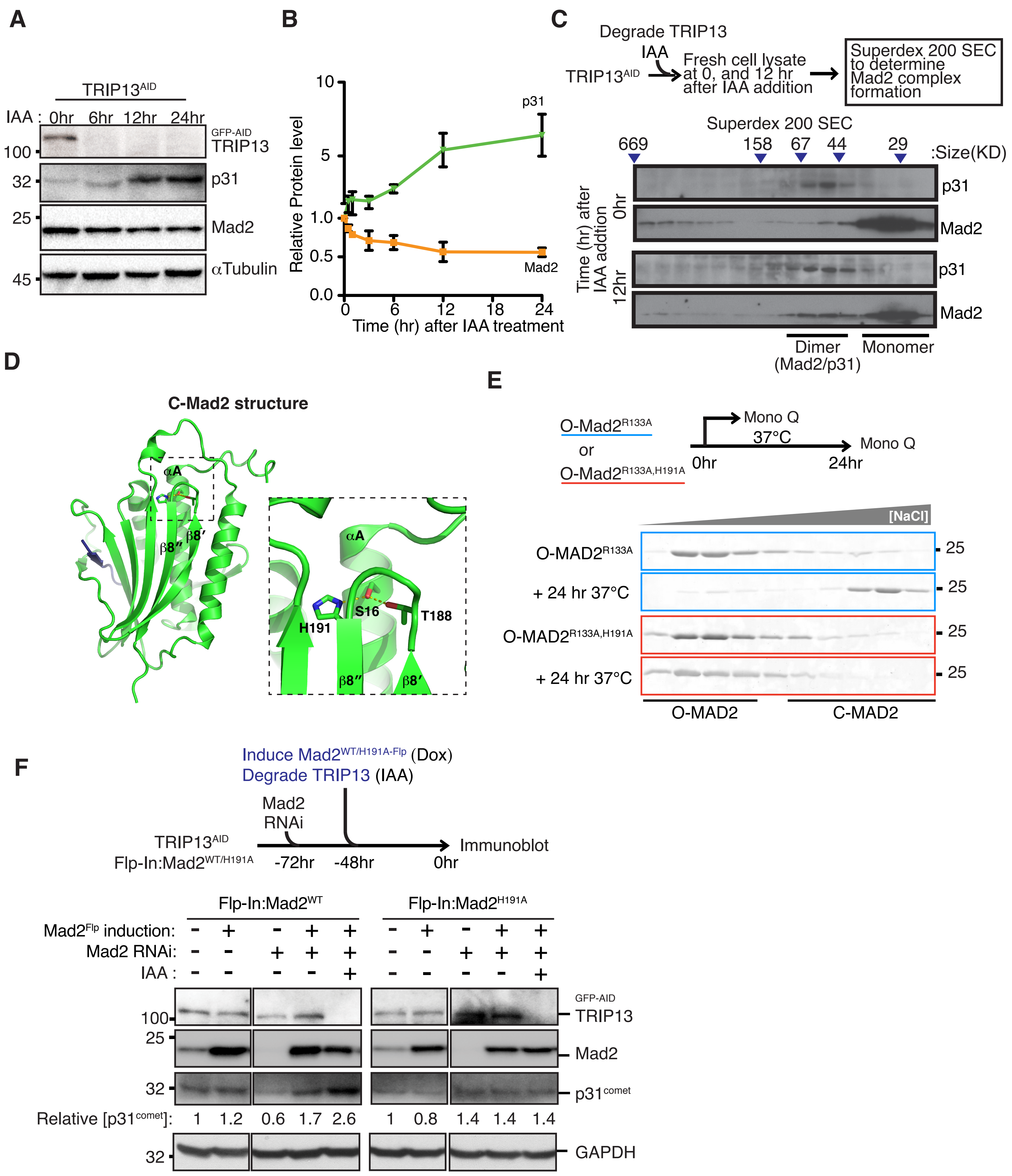

Supplementary Figure 3. TRIP13 regulates homeostasis of Mad2 and $p31^{comet}$.

A) Quantitative immunoblot analysis of Mad2 and $p31^{comet}$ levels after TRIP13 depletion in $TRIP13^{AID}$ cells.

B) Relative levels of Mad2 and $p31^{comet}$ in $TRIP13^{AID}$ cells upon TRIP13 depletion, from 3 independent quantitative immunoblot analyses.

C) Proteins from $TRIP13^{AID}$ in the presence or absence of TRIP13 were purified by size-exclusion chromatography (Superdex 200, GE Life Sciences). Fractions were collected and analyzed by SDS-PAGE followed by immunoblot against Mad2 and $p31^{comet}$ antibodies.

D) Structure of closed Mad2 (PDB ID 2V64, [16]), showing the conserved hydrogen-bond network between helix αA and strands β8' and β8'' that stabilizes the closed conformation [52, 63].

E) Mono-Q ion-exchange profiles of purified O-$Mad2^{R133A}$ (dimerization-defective mutant) versus O-$Mad2^{R133A/H191A}$, before and after a 24-hour incubation at 37°C. $Mad2^{R133A}$ converts entirely to the closed conformation, while $Mad2^{R133A/H191A}$ remains in the open conformation.

F) (Top) Schematic of the approach to replace endogenous Mad2 with $Mad2^{WT/H191A\text{-}Flp}$ in TRIP13 depleted cells. (Bottom) Endogenous Mad2 was depleted by siRNA targeting 3'UTR of Mad2 and $Mad2^{WT/H191A\text{-}Flp}$ was induced by doxycycline. Mad2 and $p31^{comet}$ levels were measured by quantitative immunoblotting. Whereas TRIP13 depletion results in a marked stabilization of $p31^{comet}$ in the presence of $Mad2^{WT}$, $p31^{comet}$ is not stabilized in the presence of $Mad2^{H191A}$, which predominantly adopts the open conformation in solution.

Supplementary Figure 4. TRIP13 and APC15 are required for turnover of interphase MCC.

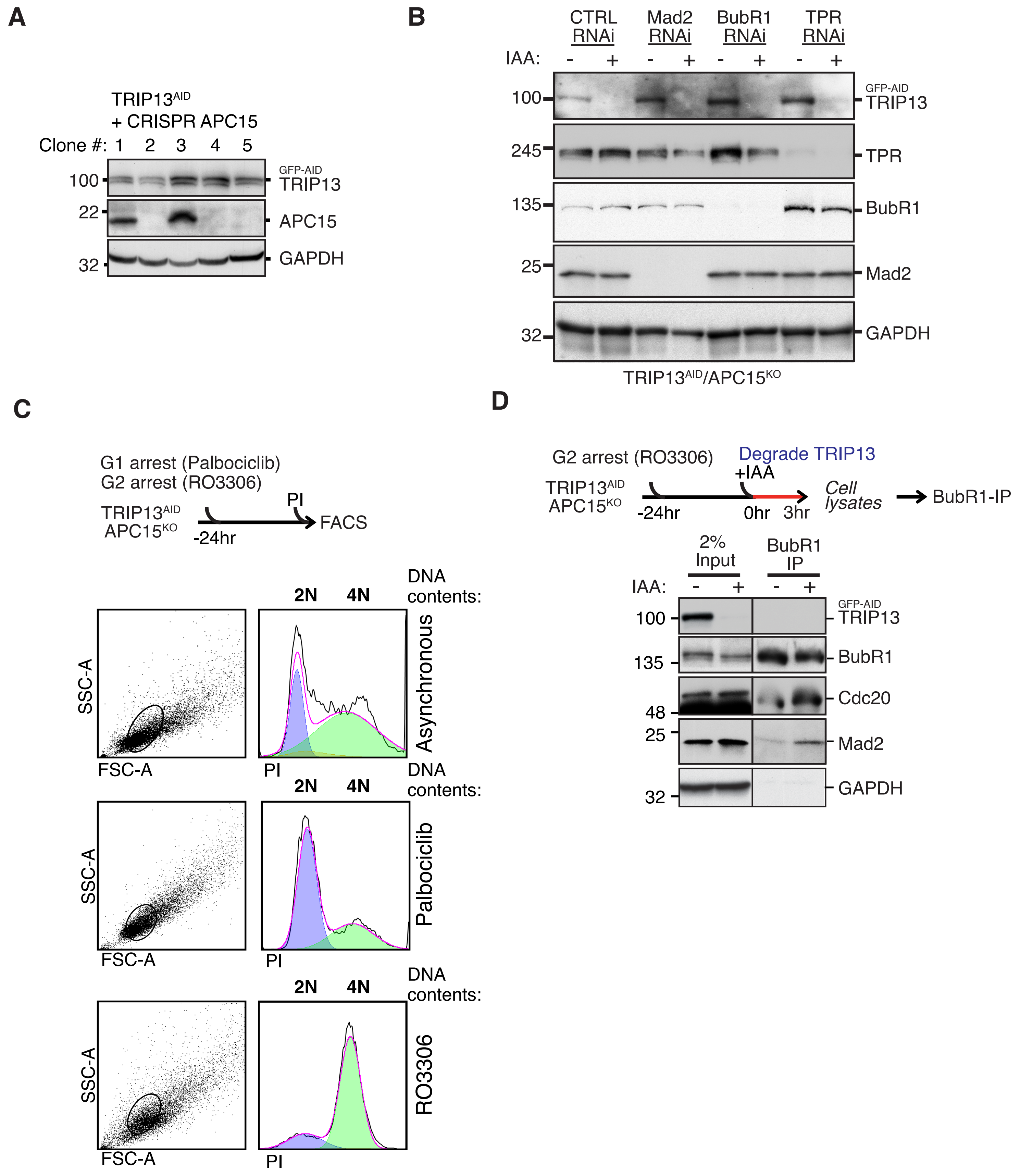

Supplementary Figure 4. Turnover of interphase MCC requires TRIP13 and APC15. (related to Figure 4)

A) Screening of TRIP13^{AID}/APC15^{KO} cells using wesern blot analysis from five independent clones, generated using CRISPR/Cas9-based gene disruption from TRIP13^{AID} cells.

B) Western blot showing effective depletion of Mad2, BubR1 and TPR by RNAi in TRIP13^{AID}/APC15^{KO} cells in Figure 4B, 4C.

C) *Upper:* Schematic of experiment to confirm synchronization of TRIP13^{AID}/APC15^{KO} cells in Figure 4D. Cells were synchronized in G1 with Palbociclib and in late G2 with RO-3306. DNA contents were measured by FACS after propidium iodide (PI) staining.

Supplementary Figure 5. Uncropped Western blot scan

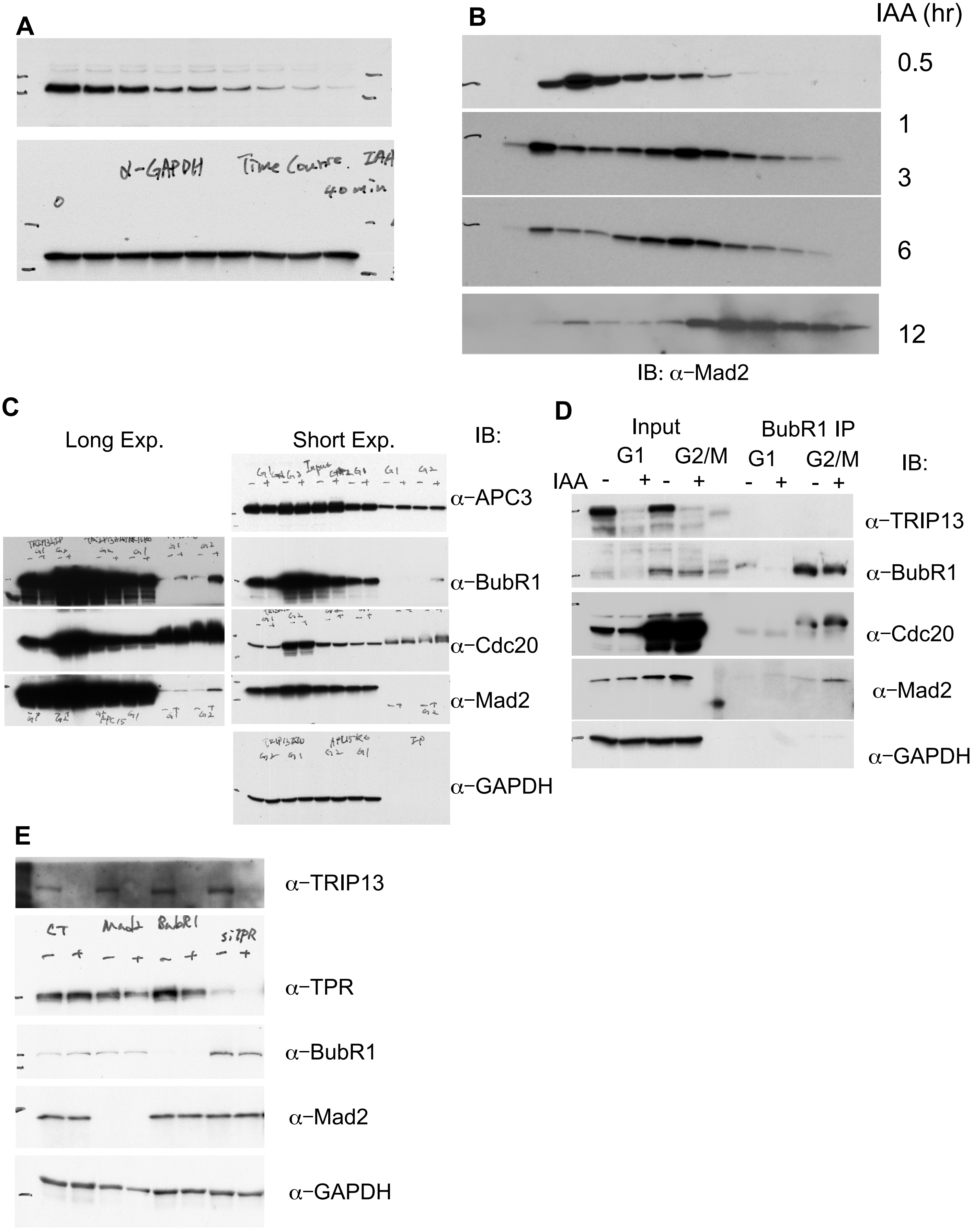

Supplementary Figure 5. Uncropped western blot scan

A) uncropped western blot scan from Figure 1B.

B) uncropped western blot scan from Figure 2A.

C) uncropped western blot scan from Figure 4D.

D) uncropped western blot scan from Supplementrary Figure 4D.
